# SNAP25 is a potential target for early stage Alzheimer’s disease and Parkinson’s disease

**DOI:** 10.1186/s40001-023-01360-8

**Published:** 2023-12-06

**Authors:** Qian Wang, Sijue Tao, Lei Xing, Jiuyu Liu, Cankun Xu, Xinyi Xu, Haohan Ding, Qi Shen, Xiaobo Yu, Yingwei Zheng

**Affiliations:** 1 Department of Radiology, Xuzhou Central Hospital, Xuzhou, 221004 Jiangsu China; 2 Laboratory Animal Center, Zhejiang University, Hangzhou, 310058 Zhejiang China; 3 Jiangsu Key Laboratory of Brain Disease and Bioinformation, Research Center for Biochemistry and Molecular Biology, Xuzhou Medical University, Xuzhou, 221004 Jiangsu China; 4 Neurological Institute, Columbia University, NY Presbyterian Hospital, New York, NY USA; 5 National Engineering Laboratory for Resource Development of Endangered Crude Drugs in Northwest of China, Shaanxi Normal University, Xi’an, 710062 Shanxi China

**Keywords:** Alzheimer’s disease, Parkinson’s disease, Bioinformatical analysis, Comorbidity mechanism, Olfactory system, SNAP25

## Abstract

**Background:**

Alzheimer’s disease (AD) and Parkinson’s disease (PD), two common irreversible neurodegenerative diseases, share similar early stage syndromes, such as olfaction dysfunction. Yet, the potential comorbidity mechanism of AD and PD was not fully elucidated.

**Methods:**

The gene expression profiles of GSE5281 and GSE8397 were downloaded from the Gene Expression Omnibus (GEO) database. We utilized a series of bioinformatics analyses to screen the overlapped differentially expressed genes (DEGs). The hub genes were further identified by the plugin CytoHubba of Cytoscape and validated in the hippocampus (HIP) samples of APP/PS-1 transgenic mice and the substantial nigra (SN) samples of A53T transgenic mice by real-time quantitative polymerase chain reaction (RT-qPCR). Meanwhile, the expression of the target genes in the olfactory epithelium/bulb was detected by RT-qPCR. Finally, molecular docking was used to screen potential compounds for the target gene.

**Results:**

One hundred seventy-four overlapped DEGs were identified in AD and PD. Five of the top ten enrichment pathways mainly focused on the synapse. Five hub genes were identified and further validated. As a common factor in AD and PD, the changes of synaptosomal-associated protein 25 (SNAP25) mRNA in olfactory epithelium/bulb were significantly decreased and had a strong association with those in the HIP and SN samples. Pazopanib was the optimal compound targeting SNAP25, with a binding energy of − 9.2 kcal/mol.

**Conclusions:**

Our results provided a theoretical basis for understanding the comorbidity mechanism of AD and PD and highlighted that SNAP25 in the olfactory epithelium may serve as a potential target for early detection and intervention in both AD and PD.

**Supplementary Information:**

The online version contains supplementary material available at 10.1186/s40001-023-01360-8.

## Introduction

Neurodegenerative diseases (NDDs) are common irreversible diseases, which are the leading causes of disability in people over 65 years [1, 2]. NDDs mainly include Alzheimer’s disease (AD), Parkinson’s disease (PD), amyotrophic lateral sclerosis (ALS), Huntington’s disease (HD), and multiple sclerosis (MS). Among them, AD and PD have higher incidences and are usually sporadic in most cases. The characteristic pathological hallmarks of AD and PD are progressive loss of neurons and accumulation of pathological proteins such as extracellular amyloid-β (Aβ) and intraneuronal neurofibrillary tangles (NFTs) in AD and α-synuclein (α-Syn) and Lewy bodies/Lewy neurites (LBs/LNs) in PD. Accumulating evidence indicates that the pathologies of AD and PD begin in the asymptomatic stage, and there are no effective therapies available to prevent or delay the progressions of AD and PD [3, 4]. Meanwhile, several studies have found that LBs are deposited in the brains of AD patients [5, 6]. Studies also proved that α-Syn was the major non-Aβ component of Aβ plaques, while NFTs presented in vulnerable brain regions of PD patients [7]. The co-occurrence of different pathologies of the two diseases suggests that AD and PD have a common molecular basis, which makes the early diagnosis and treatments of AD and PD more challenging. However, the potential common molecular basis of AD and PD remains unclear.

Besides the common pathological characteristics, similar clinical symptoms, such as cognitive disorder and olfactory dysfunction, also coexist in AD and PD patients [8, 9]. More than 90% of AD and PD patients emerged with olfactory dysfunction in the early stages. Accumulating evidence demonstrated that olfactory dysfunction occurred at Braak stages 1 and 2 of sporadic PD, with damage to olfactory structures and pathological aggregation of α-syn in the olfactory bulb in the early stage, and then spread to the brain stem and related brain regions [10–13]. Similar deposition patterns of pathological proteins can be found in AD [14, 15]. More importantly, many studies have demonstrated that olfactory dysfunction is related to cognitive impairment and preceded cognitive decline, which can be used to predict the cognitive decline of AD or PD patients [16, 17]. The olfactory system consists of the olfactory sensory neurons, the olfactory bulb (OB), and other multiple olfactory cortices. Olfactory sensory neurons in the olfactory epithelium (OE) of the nasal cavity, receive chemical information from external sources and convert the chemical signals into neural signals to the OB. The olfactory system is a complex neural network characterized by long axonal projection, with nerve fibers having neural projection connections directly or indirectly to multiple brain regions via synapse connections [18, 19]. Such structural features facilitate the diffusion of α-Syn and Aβ in the olfactory bulb to the susceptible brain regions and cause neuronal degeneration. The OB had structural–functional connections with multiple brain regions, such as the hippocampus (HIP) [14, 20–22]. The HIP is a crucial brain region charging for learning, memory, and cognition. The HIP is severely damaged in AD patients, as well as the substantial nigra (SN) in PD patients. Olfactory dysfunction is characterized by not only an increase in the olfactory threshold but also a decrease in olfactory discrimination, especially in early stage AD and PD [23, 24]. Several studies have found that pathological proteins accumulated in the OB disturbed synaptic proteins’ synthesis and induced synaptic functional dysfunction and cognitive impairment [25]. However, the common mechanism of olfactory dysfunction in AD and PD has not been fully explained yet.

Transcriptomics sequencing integrated with bioinformatics analysis has been widely applied to assay the alteration of gene expression levels and predict their possible implications in given diseases. A large amount of data are available in the authoritative public GEO database (http://www.ncbi.nlm.nih.gov/geo/). In this paper, we utilized two original gene profiles to explore the common molecular changes in AD and PD. Through a series of bioinformatics analyses, we found that the common hub genes in AD and PD mainly focused on synapse dysfunction. The mRNA expression levels of the hub genes were validated by real-time quantitative polymerase chain reaction (RT-qPCR). The odor detection and discrimination tests were used to assess the olfactory function of mice. By further molecular docking, we predicted the potential compounds for the target gene.

The present study aimed to identify the common alternations of molecules and molecular networks in early stage AD and PD and try to screen the potential targets.

## Results

### Identification of the overlapping DEGs

**Fig. 1:**
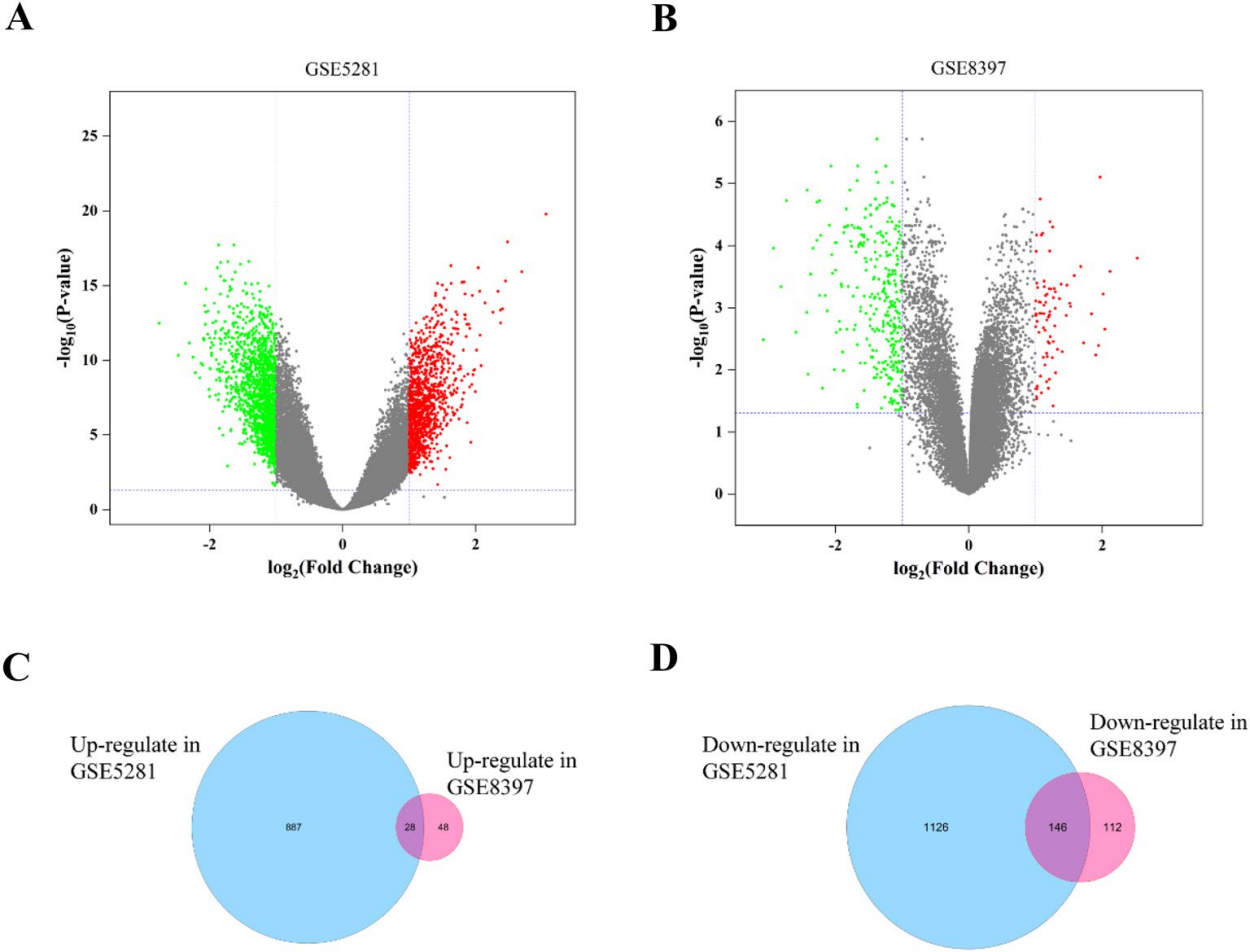
Gene expression profiles of GSE5281 and GSE8397 databases. **A** Volcano plot of gene expression profile data in GSE5281. **B** Volcano plot of gene expression profile data in GSE8397. Red dots (significantly up-regulated DEGs), blue dots (significantly down-regulated DEGs), and Grey dots (no significant changes of genes). |logFC| > 1 and adjusted *P* value < 0.05 were as cutoff threshold. **C** Venn diagram of the up-regulated overlapping DEGs. **D** Venn diagram of the down-regulated overlapping DEGs

The gene expression profiles of GSE5281 (AD) and GSE8397 (PD), were used to identify overlapping DEGs in AD and PD brain samples. twofold change and *P* < 0.05 were as set as the cutoff criteria. A total of 2159 genes (915 up-regulated genes and 1244 down-regulated genes) were extracted from the GSE5281 data set (Fig. 1A), and 334 genes (76 up-regulated genes and 258 down-regulated genes) from the GSE8397 data set (Fig. 1B). We compared the up-regulated and down-regulated DEGs of GSE5281 and GSE8397, respectively, and finally identified 28 overlapping up-regulated DEGs and 146 overlapping down-regulated DEGs as shown in the Venn diagram (Fig. 1C, D). The 174 overlapping DEGs were used in the subsequent analysis.

### Enrichment analysis of the overlapping DEGs

**Fig. 2:**
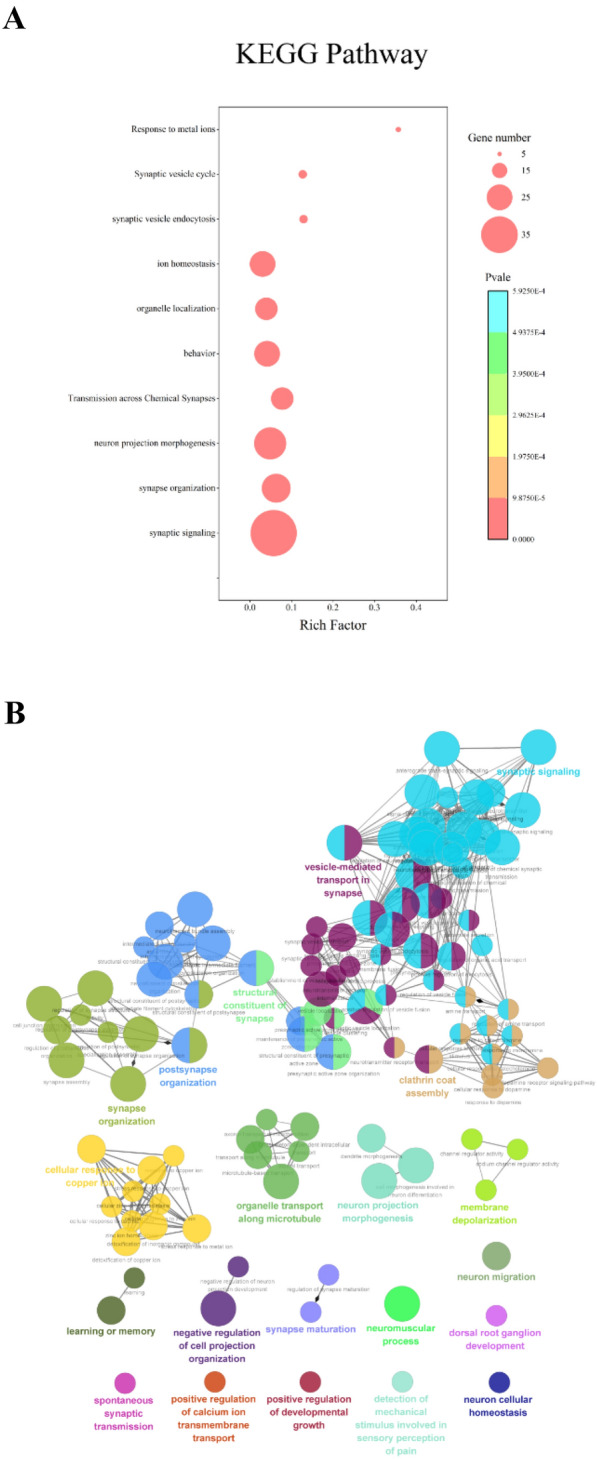
Enrichment analyses of the overlapping DEGs in GSE5281 and GSE8397 databases. **A** GO enrichment analysis of the overlapping DEGs. The *X*-axis represents the *q* value (− log10), and the *Y*-axis represents the GO terms. The GO terms were measured by the rich factor, *q* value, and number of genes enriched. The greater the Rich factor is, the greater the degree of enrichment and the greater the *P* value. The brighter the color of red is, the more significant the term. **B** ClueGO analysis of the overlapping DEGs from AD/PD patients. Functionally grouped network with terms as nodes linked based on their kappa score level (≥ 0.4), where only the label of the most significant term per group is shown. The node size represents the term enrichment significance. Functionally related groups partially overlap

GO and KEGG enrichment terms of the overlapping DEGs were obtained with a *P* value < 0.01, using Metascape and the plugin ClueGo of Cytoscape. The top 10 enrichment terms were selected and shown in Fig. 2A. Notably, five terms out of the top 10 terms including synaptic signaling, synapse organization, transmission across chemical synapses, synaptic vesicle endocytosis, and synaptic vesicle cycle in the synapse were relative to synaptic structure and function. As shown in Fig. 2B, vesicle-mediated transport in synapses, spontaneous synaptic transmission, synaptic signaling, post-synapse organization, synapse maturation, and structural constituent of synapse had high correlations with synaptic structure and function. Taken together, these results revealed that overlapping DEGs mainly focused on synaptic dysfunction in the occurrence of AD and PD.

### Construction of the PPI network and selection of hub genes

**Fig. 3:**
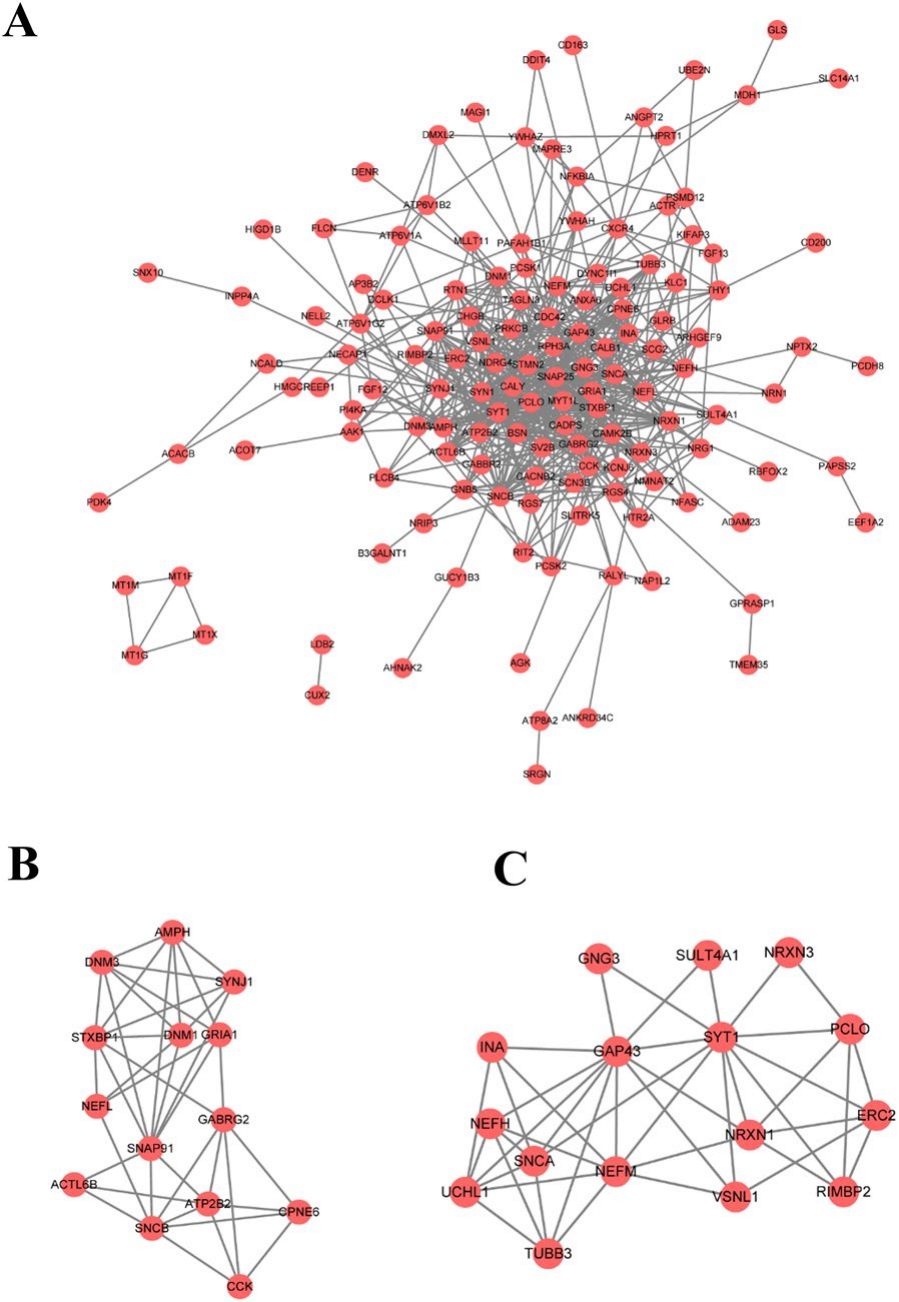
PPI network and modular analysis of the overlapping DEGs. **A** PPI network was constructed by a total of 174 DEGs via the STRING database, and visualized by Cytoscape. The nodes represent proteins, the edges represent the interaction of the proteins. **B**, **C** Top two PPI clusters in MCODE analysis. degree cutoff = 2, node score cutoff = 0.2, k-core = 2 and max. Depth = 100

The overlapping DEGs were used to construct a PPI network via the String online and visualized via Cytoscape (Fig. 3A). The top two clusters with the highest clustering scores were obtained via MCODE. The first cluster had 6000 scores, 14 nodes, and 39 edges (Fig. 3B), including SNAP91, DNM3, DNM1, STXBP1, NEFL, AMPH, SYNJ1, GRIA1, GABRG2, ATP2B, CPNE6, CCK, SNCB, and ACTL6B. The second one had 5733 scores, 16 nodes, and 43 edges (Fig. 3C). Then, the PPI network was re-analyzed with the CytoHubba plugin to select the top 10 genes according to the MCC, MNC, and degree topological algorithm, respectively (Table 1). Finally, we obtained five overlapping hub genes after three Algorithms’ Comparison, including synaptosomal-associated protein 25(SNAP25), synapsin 1 (SYN1), synaptotagmin1 (SYT1), growth-associated protein 43 (GAP43) and synaptosomal-associated protein 91 (SNAP91) (Table 1). Collectively, the five hub genes encoded all presynaptic proteins and were both down-regulated in AD and PD patients.

**Table 1:** Top 10 genes screened by three algorithms in CytoHubba

MCC	MNC	Degree	The overlapping genes
**SNAP25**	**SNAP25**	**SNAP25**	**SNAP25** **SYN1** **SYT1** **GAP43** **SNAP91**
**SYN1**	**SYN1**	**SYT1**	
**SYT1**	**SYT1**	**SYN1**	
**SNAP91**	**GAP43**	**GAP43**	
STXBP1	**SNAP91**	GABRG2	
DNM1	NRXN1	STMN2	
SYNJ1	SNCB	SNCB	
**GAP43**	STMN2	NRXN1	
AMPH	GRIA1	**SNAP91**	
DNM3	NEFL	NEFL	

The bolded genes represent genes that are coexisting in all three algorithms

*MCC* the maximal clique centrality, *MNC* maximum neighborhood component

### Validation of representative hub genes by real-time qPCR in 6-month AD and PD mice

**Fig. 4:**
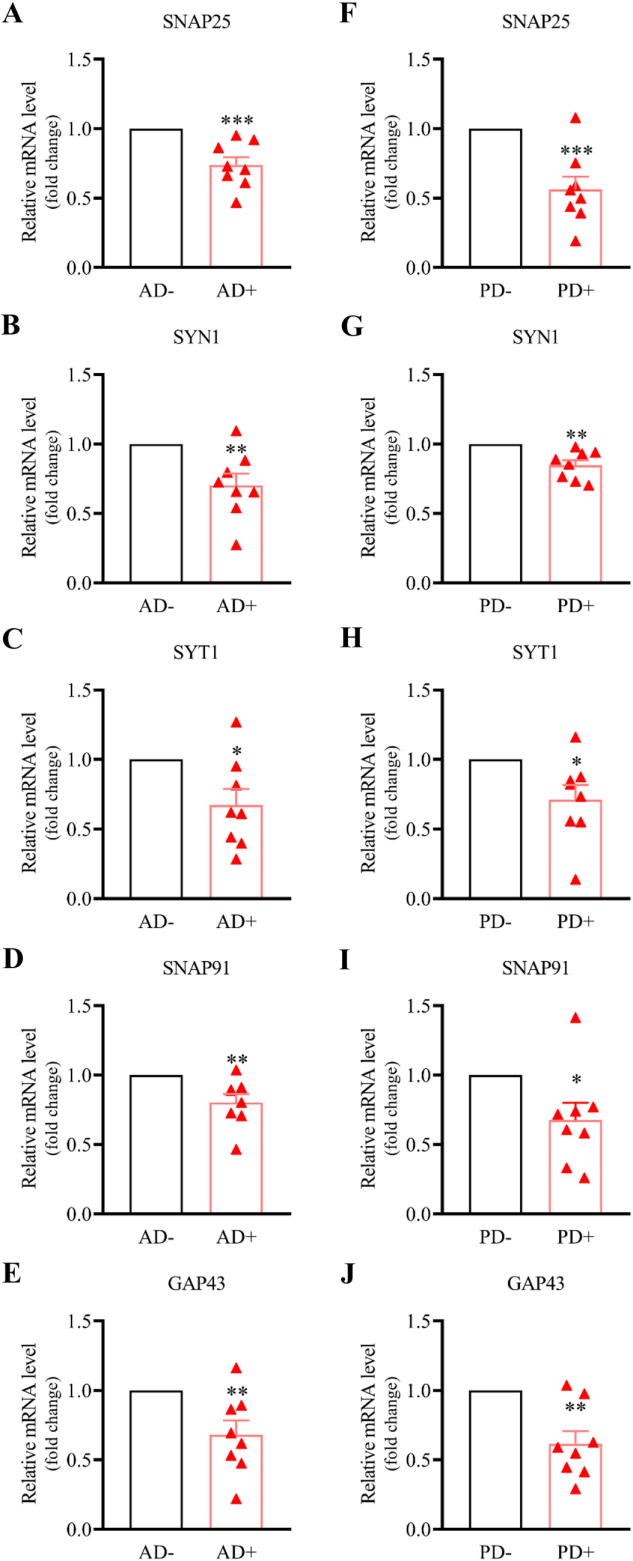
Validation of five hub genes in 6-month AD and PD mice samples. The mRNA levels of five hub genes were validated in 6-month AD+/PD+ mice samples and age-matched control mice samples by real-time qPCR. Five hub genes include VAMP2, SNAP25, SYT1, SYN1, NSF, and STX1A. All hub genes significantly declined in both AD+/PD+ mice samples, compared to those in control samples (AD− and PD−). Relative quantification (RQ) values were calculated. Data are the mean ± s.e.m from 8 mice in each group. A *T* test was used to analyze the difference between the two groups. *0.01 ≤ *P* < 0.05, **0.001 ≤ *P* < 0.01, ***P < 0.001

We used RT-qPCR to validate the expression of the five hub genes in 6-month AD and PD mice and their age-matched control mice separately. As shown in Fig. 4A–E, we found that the expression level of all the selected hub genes was more significantly decreased in AD+ mice than in AD− mice (SNAP25, 0.74 ± 0.15, *P* = 0.0005; SYN1, 0.70 ± 0.23, *P* = 0.0037; SYT1, 0.67 ± 0.30, *P* = 0.0129; GAP43, 0.68 ± 0.27, *P* = 0.0079; SNAP91, 0.80 ± 0.16, *P* = 0.0064). Similarly, we also found that the expression level of all the hub genes was more significantly decreased in PD+ mice (SNAP25, 0.56 ± 0.25, *P* = 0.0004; SYN1, 0.85 ± 0.10, *P* = 0.0010; SYT1, 0.71 ± 0.28, *P* = 0.0166; GAP43, 0.62 ± 0.25, *P* = 0.0010; SNAP91, 0.68 ± 0.33, *P* = 0.0210, Fig. 4F–J). To explore the significance of the hub genes, we used ANOVA to detect hub genes separately. We found that the expression of SNAP25 and SNAP91 were most significant in the HIP in AD mice, while SNAP25 and GAP43 in the SN with PD mice (AD: *P*_SNAP25_ = 4.11E−03, *P*_GAP43_ = 2.18E−13; PD: *P*_SNAP25_ = 0.017, *P*_SNAP91_ = 0.031). All above, the results revealed those synapse-relative hub genes had changed in 6-month AD and PD mice. SNAP25, as a common hub gene in AD and PD, has the most significant changes.

### Olfactory deficit in the 6-month AD and PD mice

**Fig. 5:**
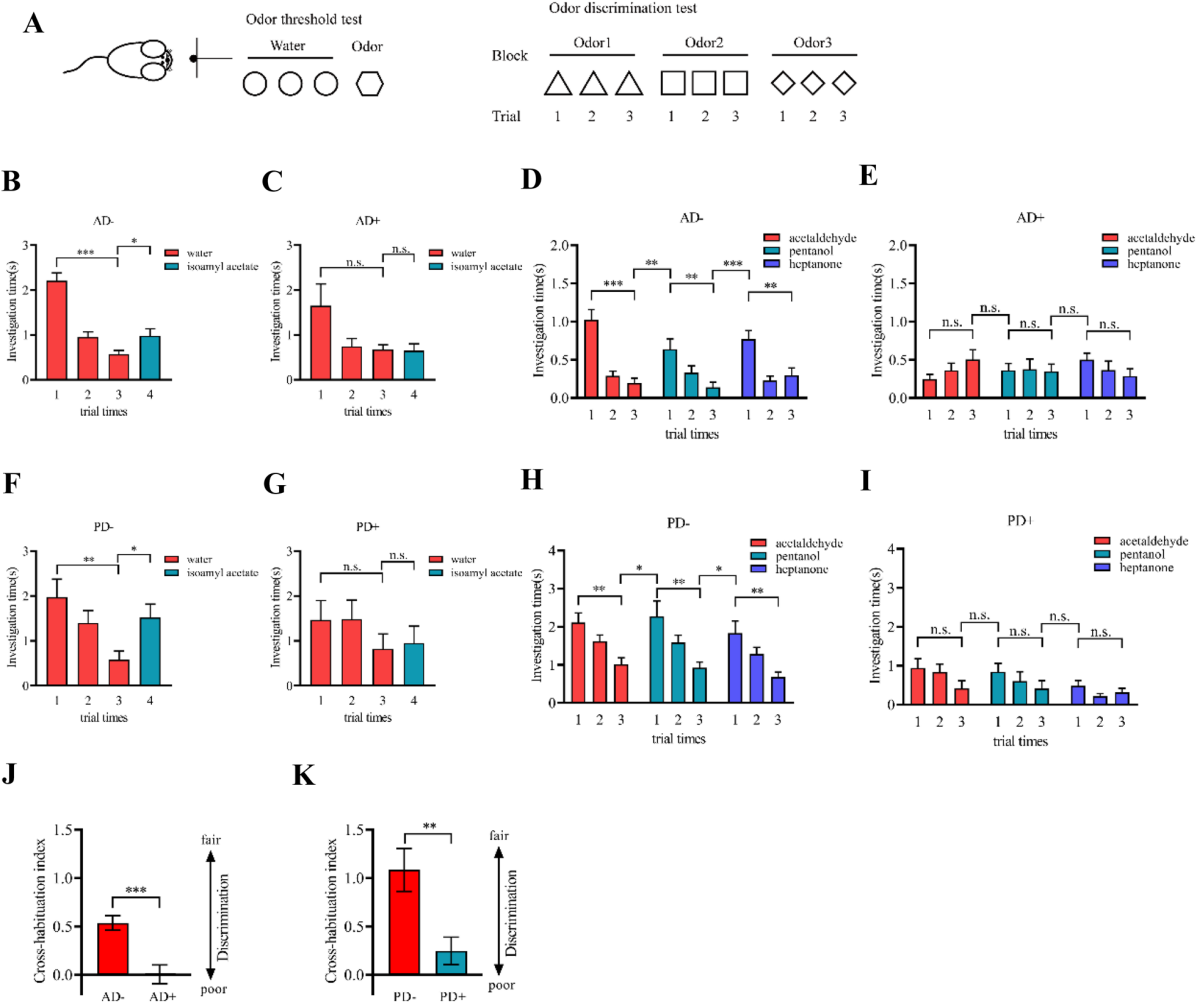
6-month AD and PD mice showed olfactory defects. **A** Behavioral diagram of odor detection and odor discrimination. **B**, **C** Exploring times of AD+ mice were decreased than those of AD- mice in the odor detection task. **D**, **E** Exploring time of AD+ and AD− mice in odor discrimination task. **F**, **G** Exploring times of AD+ mice were decreased than that of AD− mice in the odor detection task. **H**, **I** Exploring time of AD+ and AD− mice in odor discrimination task. **J**, **K** Discrimination index of AD and PD mice declined. Data are the mean ± s.e.m from 8 mice in each group. A *T* test was used to analyze the difference between the two groups. *0.01 ≤ *P* < 0.05, **0.001 ≤ *P* < 0.01, ***P < 0.001

The olfactory dysfunction of mice was evaluated using odor detection and odor discrimination tests. The procedures are shown in Fig. 5A. Previous studies had demonstrated that AD or PD mice at 6-month-old showed abnormal aggregation of pathological proteins and atypical clinical symptoms [26–28]. Thus, the 6-month-old mice in the early stages of AD and PD were used in these olfactory function tests. It is well-known that the mice exhibit habituation for the same odor, while dishabituation for the novel odor. During the odor detection test, AD− mice exhibited normal habituation for the same odor and an increase in exploring time for novel odor (*P* < 0.05, Fig. 5B). However, the exploring time of AD+ mice showed no obvious change for the same or novel odors (*P* > 0.05, Fig. 5C). Similar results were found in 6-month PD− and PD+ mice (Fig. 5F, G). During the odor discrimination test, AD− mice showed significant habituation for the same odor, and an obvious dishabituation for novel odor (*P* < 0.05, Fig. 5D). Conversely, AD+ mice did not display the habituation for the same odor nor dishabituation for similar and novel odor (*P* > 0.05, Fig. 5E). A similar pattern was also found in PD− and PD+ mice (Fig. 5H, I). In addition, the cross-habituation indexes of AD and PD mice were calculated based on the odor discrimination tests. Compared to AD− mice, the cross-habituation index of AD+ mice robustly declined, as well as that in PD+ mice (all *P* < 0.01, Fig. 5J, K).

### Correlation analysis of the SNAP25 mRNA between the OB and OE samples and the HIP or SN samples

**Fig. 6:**
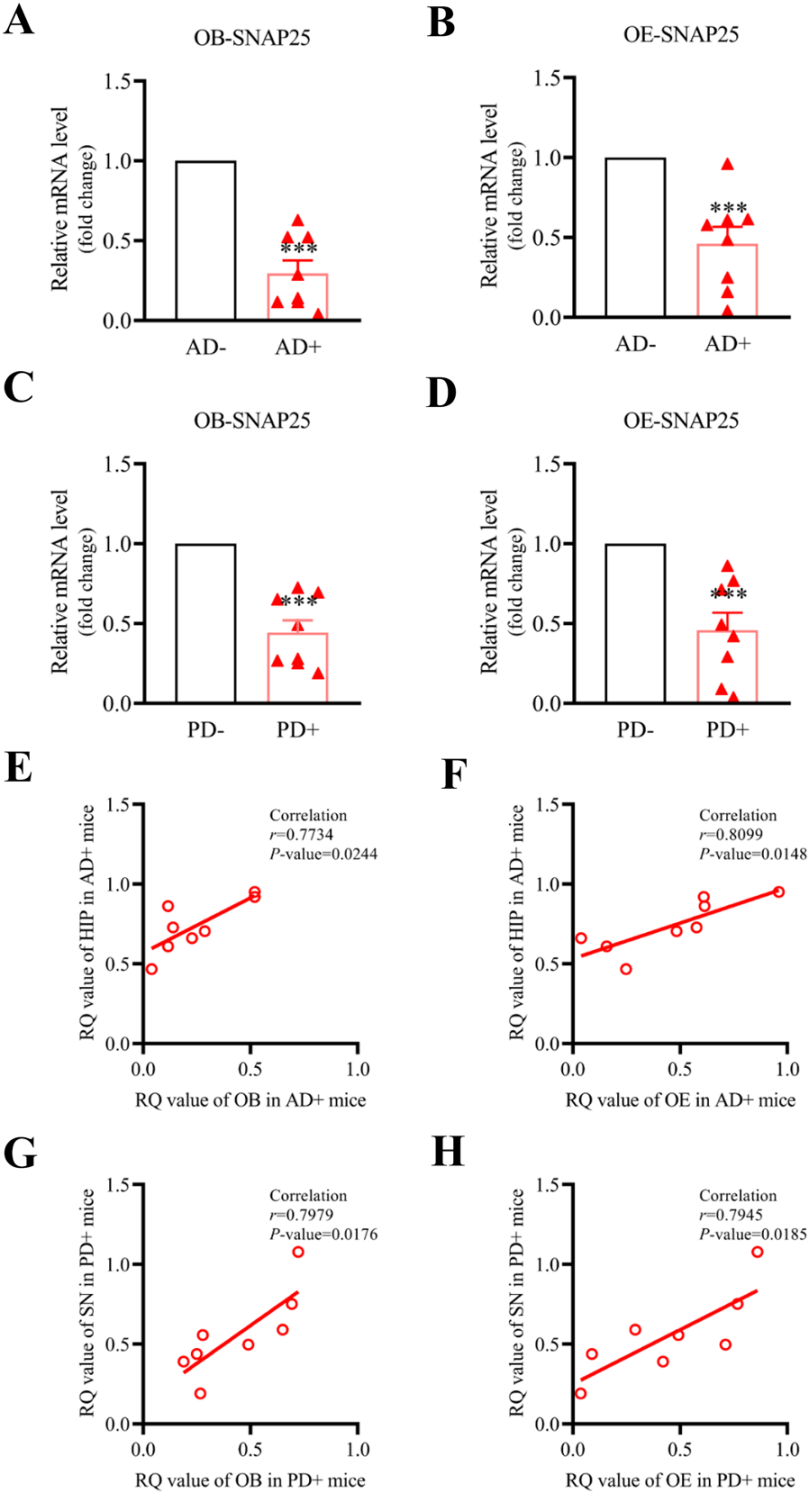
Correlation of expression of SNAP25 between the OB/OE and HIP/SN samples. **A**, **B** Real-time qPCR displayed that the expressions of SNAP25 in the OB and OE were significantly declined in 6-month AD+ mice samples than those in AD- mice samples. **C**, **D** Real-time qPCR revealed the expressions of SNAP25 in the OB and OE decreased in 6-month PD+ mice samples than those in PD- mice samples. **E**, **F** Pearson correlative analysis revealed that SNAP25’s expressions in the OE and OB have a high correlation with those in the HIP in AD+ mice. **G**, **H** Pearson correlative analysis revealed that SNAP25’s expressions in the OE and OB have a high correlation with those in the HIP in PD+ mice. Expression of SNAP25 was normalized by β-actin. Relative quantification (RQ) values were calculated. Data are the mean ± s.e.m from 8 mice in each group. A *T* test was used to analyze the difference between the two groups. *0.01 ≤ *P* < 0.05, **0.001 ≤ *P* < 0.01, ***P < 0.001. Pearson correlative analysis was used to analyze the correlation of SNAP25 expression between the OB/OE and the HIP/SN

To define the changes of SNAP25 in the OB and OE of AD and PD mice, we used real time-qPCR to validate the expression levels of SNAP25 in 6-month AD+ and PD+ mice and age-matched AD− and PD− mice separately. As shown in Fig. 6A, B, we found that the expression of SNAP25 decreased both in the OB and OE of AD+ mice compared to AD− mice (in OB, 0.30 ± 0.21, *P* < 0.0001; in OE, 0.46 ± 0.28, *P* = 0.0001). Similarly, we found that the expression of SNAP25 in the OB and OE significantly decreased in PD+ mice compared to PD− mice (in OB, 0.44 ± 0.20, *P* = 0.0001; in OE, 0.45 ± 0.28, *P* = 0.0002, Fig. 6C, D). In addition, we correlated the RQ values of SNAP25 in the HIP/SN with those in the OB and OE of AD and PD mice. As shown in Fig. 6E, F, they exhibited a high correlation (HIP vs OB, *r* = 0.7734,* P* = 0.0244; HIP vs OE, *r* = 0.8099,* P* = 0.0148). Similar results were found in PD mice (SN *vs* OB, *r* = 0.7979,* P* = 0.0176; SN vs OE, *r* = 0.7945,* P* = 0.0185, Fig. 6G, H). Our results showed that the expressions of SNAP25 in the OB and OE decreased in the early stages in AD and PD mice and were highly correlated with those in representative brain areas of AD and PD mice.

### Virtual screening of potential compounds targeting SNAP25

**Fig. 7:**
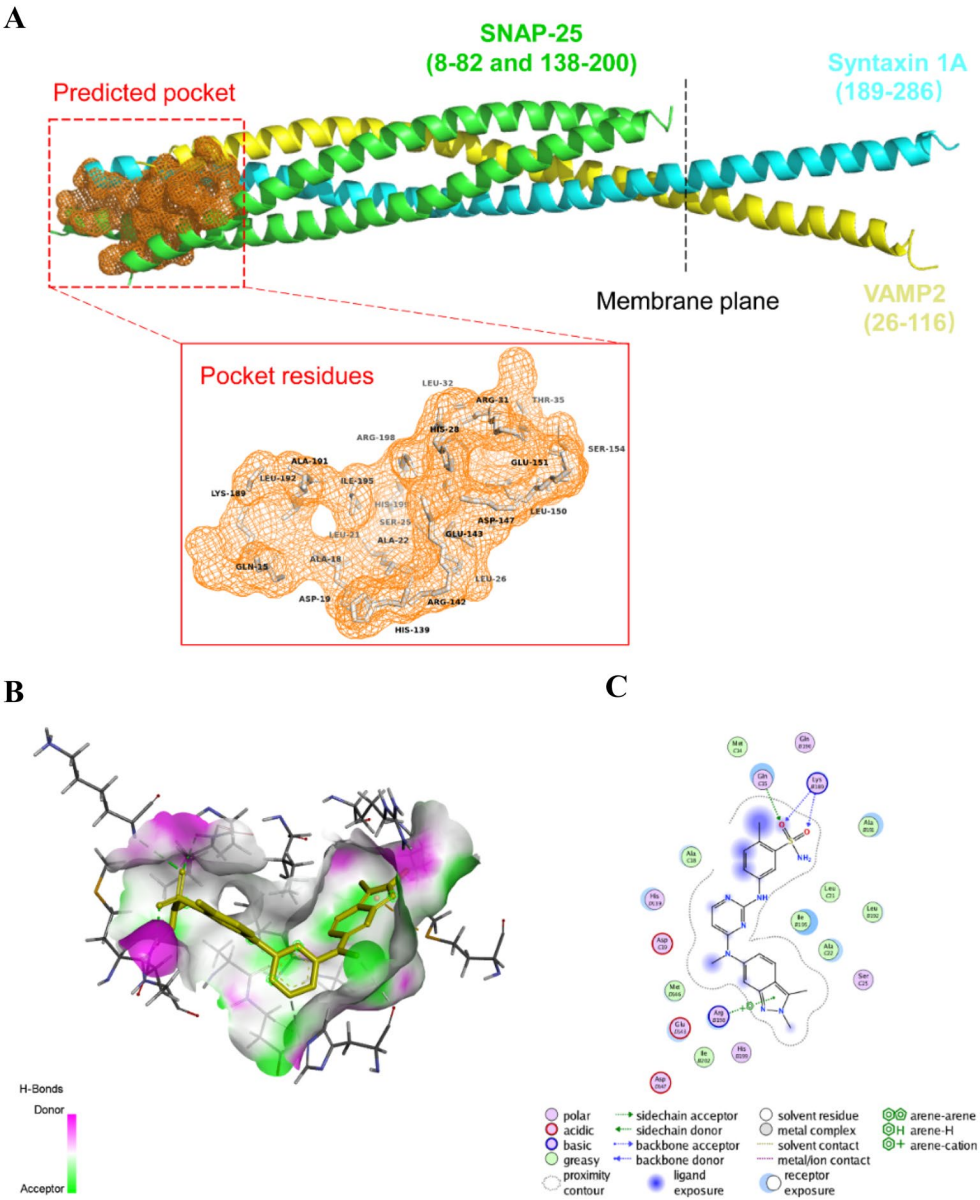
Virtual screening results between SNAP-25 and FDA-approved drugs. **A** Structure of the rat neuronal SNARE complex (PDB accession#: 3HD7^[22]^ and binding pocket predicted by PROTEINS PLUS (https://proteins.plus/). **B** H-bond receptor surface between SNAP-25 and Pazopanib. **C** Residues of interaction between Pazopanib and SNAP-25. The other corrected information of virtual screening is shown in Table 2

SNAP25 is a part of the soluble *N*-ethylmaleimide-sensitive fusion protein attachment protein receptor (SNARE) complex which is critical for synaptic vesicle fusion in the presynaptic membrane, a key step in the release of neurotransmitters. SNAP25 is composed of 206 amino acid residues. When synaptic vesicles are close enough to the presynaptic membrane, VAMP, syntaxin, and the cytoplasmic region of SNAP25 bind to each other to form a tight complex. In addition, SNAP25 interactions with SYT1, take part in the mechanism of membrane fusion initiated by Ca^2+^, and are involved in the fusion pore dynamics regulating the final steps of transmitter release [29]. SNARE functional interfaces are usually large and extended, making it difficult for small molecules to modify their conformation significantly. In the present study, we screened small-molecule drugs targeting SNAP25 via virtual screening. Based on the crystal structure of SNAP25, we first predict its potential functional pockets (Fig. 7A). On top of that, ten computationally plausible small-molecule drugs were captured according to their binding energies. The specific information on the residues of drug–protein interactions was listed (Table 2). Among them, Pazopanib with a binding energy of − 9.2 kcal/mol was the best-scoring drug. Moreover, Pazopanib was able to perfectly embed in the predicted activity pocket and interacted with SNAP25 protein residues at ALA18, HIS139, GLU143, ILE195, MET14, GLN15, ALA18, LYS189, ALA191, LEU192, and ARG198 via van der Waals interactions and hydrogen bonding (Fig. 7B, C). These results suggested that SNAP25 could be used as a potential early intervention target for AD and PD.

**Table 2:** Docking score and predicted protein–ligand interaction of the top ten compounds selected in virtual screening

Drug name	Molecular formula	Docking Score (kcal/mol)	Noncovalent interactions	Residues
Pazopanib	C_21_H_23_N_7_O_2_S	− 9.2	6 Hydrophobic interactions, 7 H-bonds	ALA18C, HIS139D, GLU143D, ILE195B, **MET14C, GLN15C, ALA18C, LYS189B, ALA191B, LEU192B, ARG198B**
S-apomorphine	C_17_H_17_NO_2_	− 9.1	8 Hydrophobic interactions, 1 H-bond, 1 salt bridge	ALA22C, ARG142D, GLU143D, ILE195B, ARG198B, **SER25C,** GLU143D
Paliperidone	C_23_H_27_FN_4_O_3_	− 8.9	5 hydrophobic interactions, 1 H-bond	GLN15C, GLU143D, ILE195B, ARG198B, **HIS139D**
Cromolyn	C_23_H_16_O_11_	− 8.9	6 Hydrophobic interactions, 4 H-bonds, 1 salt bridge	ALA18C, ASP19C, GLU143D, ALA191B, ILE195B,** GLN15C, LEU21C, ARG142D, HIS199B**
Risperdal	C_23_H_27_FN_4_O_2_	− 8.8	5 Hydrophobic interactions, 1 H-bond	ALA18C, GLU143D, ALA191B, ILE195B, ARG198B, **HIS139D**
Troglitazone	C_24_H_27_NO_5_S	− 8.8	7 Hydrophobic interactions, 1 H-bond	ALA18C, ALA22C, HIS139D, GLU143D, ALA191B, LEU192B, ILE195B, **ARG198B**
Fanapt	C_24_H_27_FN_2_O_4_	− 8.8	6 Hydrophobic interactions, 3 H-bonds	ALA18C, ALA22C, GLU143D, ILE195B, **LYS189B, ALA191B, LEU192B**
Cinacalcet	C_22_H_22_F_3_N	− 8.8	8 Hydrophobic interactions, 1 H-bond	GLN15C, ALA18C, GLU143D, ILE195B, ARG198B, **HIS139D**
Alaway	C_23_H_23_NO_5_S	− 8.7	4 Hydrophobic interactions, 1 H-bond	ALA22C, ARG142D, GLU143D, ILE195B, **ALA18C**
Zelboraf	C_23_H_18_ClF_2_N_3_O_3_S	− 8.7	5 Hydrophobic interactions, 3 H-bonds, 1 halogen bond	ALA18C, ALA22C, ALA191B, ILE195B, **ASP19C, GLU143D, ARG198B, LYS189B**

Bold indicates amino acid residues that form hydrogen bonds with the active center

## Discussion

In this study, we identified common gene expression profiles in early stage AD and PD via a publicly available GEO database and found five common core molecules associated with synapse in AD and PD data sets, including SNAP25, SYN1, SYT1, GAP43, and SNAP91. All these hub genes were downregulated in AD and PD patient samples. SNAP25 significantly decreased in the HIP of 6-month AD mice and the SN of 6-month PD mice, as well as in the OE and OB samples of both types of mice. Moreover, the decline in the OE and OB displayed a strong association with those in the HIP and SN samples. Through molecular mocking, we further predicted the potential compounds targeting SNAP25. These data suggest that SNAP25 may be a potential target for early intervention in AD and PD.

In the present study, we identified 174 overlapping DEGs (28 up-regulated and 146 down-regulated) as common DEGs in two data sets. In enrichment analysis against GO terms and KEGG pathways, we found that five of the top 10 GO terms were relative to synaptic structure and function (Fig. 2A). Similar results can also be found in ClueGo enrichment analysis (Fig. 2B). Our results suggest that synaptic dysfunction was involved in the early occurrence of AD and PD, consistent with previous studies showing that synaptic dysfunction occurred in AD and PD, even in the asymptomatic phase [30, 31]. Moreover, we obtained 5 common hub genes (SNAP25, SYN1, SYT1, GAP43, SNAP91) in AD and PD databases. All of them expressed presynaptic proteins and played crucial roles in synaptic formation and neurotransmitter release. Like those in AD and PD data sets, the mRNA level of five hub genes significantly declined in HIP samples of early stage AD mice and SN samples of PD mice (Fig. 4A–J). Among them, the change of SNAP25 was the most obvious. Previous studies found that the genetic polymorphism of SNAP25 was correlated with AD and PD progression [32, 33]. In AD and PD patients, the decline of SNAP25 levels in the cortex caused neuronal degeneration [34]. In AD patients with mild cognitive impairment, SNAP25 was elevated in cerebrospinal fluid and had a strong association with t-tau [35, 36].

Olfactory dysfunction is a common symptom of AD and PD [23, 37]. More than 90% of AD and PD patients had early onset olfactory dysfunction. Olfactory dysfunction precedes cognitive decline, which reflects the deterioration in the cognitive function of patients [24]. Several studies have found that pathological proteins accumulated in the OB induced synaptic dysfunction and cognitive impairment [25]. In the present study, we validated the expression of SNAP25 in several samples across multiple brain regions of AD and PD mice. Interestingly, SNAP25 decreased in the OB and OE samples, as well as in the HIP and SN samples (Figs. 3A, F; 5A–D). Moreover, the expressions of SNAP25 in the OB and OE were strongly correlated with those in the HIP of AD mice and the SN of PD mice samples (Fig. 5E–H). Several studies have demonstrated that SNAP25 in cerebrospinal fluid was a potential synaptic biomarker, and was associated with cognitive decline in AD and PD patients [35, 38, 39]. Considering that the OE is localized in the nasal cavity, it is convenient to collect test samples or pursue drug inventions in the early stages of AD and PD. In the present study, we predicted the potential compounds bonded with SNAP25 via virtual screening (Fig. 7 and Table 2). Thus, it suggests that SNAP25 in the OE and OB may be a potential drug target for the prevention of AD and PD progression.

## Conclusion

A drawback of this paper is that even though we identified SNAP25 as a potential and available drug invention target for AD and PD, further validation of the effectiveness and sensitivity of SNAP25 as a potential target in the OE is still lacking. Meanwhile, the predicted activation center of SNAP25 was originally from the SNARE complex, due to the absence of a separate crystal structure for SNAP25. Thus, we are aware that SNAP25 as a potential target of AD and PD needs a large number of clinical samples and both in vivo and in vitro experiments for further validation.

In summary, we here highlighted the potential comorbidity mechanism of AD and PD and identified the synaptic protein SNAP25 as a potential common target for AD and PD. Furthermore, we also predicted the potential compounds targeting SNAP25 for possible early intervention in AD and PD.

## Materials and methods

### Animals

AβPP/PS-1 mice (AD) were purchased from the Beijing HuFuKang bioscience company (Beijing, China). A53T transgenic mice (PD) were purchased from Shanghai Model Organisms Center, Inc. Experiments were performed on 6-month AD+ and PD+ mice, and the corresponding control mice (AD− and PD−). The animals were housed on a 12/12 light/dark cycle with ambient temperature (21 ± 1 °C), humidity (50 ± 5%), and food and water ad libitum. All operations and procedures were approved by the animal committee of Xuzhou Medical University.

### Data set selection and preprocessing

The gene expression profiles of GSE5281 and GSE8397 were downloaded from the Gene Expression Omnibus (GEO) database. There are 87 late-onset AD patients’ data and 74 age-matched control subjects’ data in the GSE5281 (GPL570) data set. GSE8397 (GPL96) data sets contain 30 PD patients’ data and 17 age-matched control patients’ data. We used GEO2R (https://www.ncbi.nlm.nih.gov/geo/geo2r/), an interactive online tool, to identify differentially expressed genes (DEGs). With default settings, the adjusted *P* values were selected to decrease the false positive rate using the Hochberg false discovery rate and the Benjamini method. A *P* value < 0.05 and an absolute log fold-change (FC) greater than 1 for the DEGs were used as the cutoff criteria. Up/down-regulated overlapping DEGs in the GSE5281 and GSE8397 data sets were identified by Venn Diagrams. The DEGs were visualized by volcano maps using OrginPro2021b.

### Functional enrichment analysis of the overlapping DEGs

Gene ontology (GO) and Kyoto Encyclopedia of Genes and Genomes (KEGG) pathway enrichment analysis were performed to predict the biological functions of DEGs. In the present study, Metascape (http://metascape.org/) and the plugin ClueGo of Cytoscape were used to perform functional enrichment analysis of the overlapping DEGs with a *P* value < 0.01. The top 10 items were selected to show the possible functional role of the overlapping DEGs. In the calculated process of ClueGo, a kappa coefficient was calculated to reflect the functional correlations between paths or terms based on gene overlaps between pathways or GO terms. The kappa threshold defaulted to 0.4. Functionally similar entries were displayed in the same color. The threshold for enrichment significance was *P* < 0.05. The results of the enrichment analysis of the overlapping DEGs were visualized by bubble diagrams.

### PPI network construction of the overlapping DEGs and Hub gene selection

The PPI network of the overlapping DEGs was constructed using the STRING online (http://string-db.org/), which predicted experimental interactions of proteins [40], with a confidence score ≥ 0.4 for significant differences. The PPI network was visualized by Cytoscape (URL). Molecular complex detection (MCODE), a plugin of Cytoscape, was used to obtain core modules of the PPI network. The screening threshold was set at 2 as the degree cutoff, 0.2 as the node score cutoff, 2 as the k-core, and 100 as the max depth. The top two clusters were obtained as final core modules. Furthermore, the PPI network was re-analyzed by the CytoHubba plugin of Cytoscape software (version 3.7.2). The top 10 hub genes were selected from three algorithms, including the maximal clique centrality (MCC), maximum neighborhood component (MNC), and degree. Finally, five overlapping hub genes were obtained as final hub genes.

### RNA extraction and RT-qPCR

Fresh tissues of the HIP, SN (ventral dorsal midbrain), OB, and OE were obtained, respectively, from 6-month AD and PD mice and age-matched wild-type mice. Total RNA was extracted using a TRIzol reagent (KeyGen, Nanjing, China). Reverse transcription of total RNA and qPCR were performed according to the manufacturer’s recommendations of the reagent kit (MCE, New Jersey, USA). The primers are shown in Additional file 1: Table S1. All primers were used in the HIP and SN, and only the SNAP-25 primer was used in the OB and OE samples. Relative quantification (RQ) values were calculated using the 2^−ΔΔCt^ method [41]. RQ values of the hub genes were calculated based on the ratio between RQ values of hub genes in AD and PD mice and normalization to β-actin in the control group.

### Olfactory behavior test

The odor detection and odor discrimination tests were used to test the olfactory function of mice. Isoamyl acetate (diluted 1 × 10^–6^ in paraffin oil) was used in the odor detection test. Meanwhile, acetaldehyde, pentanol, and heptanone (diluted 1 × 10^–3^ in paraffin oil) were used in the odor discrimination test. Before the experiment started, the mice were habituated in the testing cage with a cotton applicator for 3 min. During the odor detection experiment, the mice received three successive trials of water followed by one trial of isoamyl acetate. Each trial lasted for 1.5 min, with an inter-trial interval of 30 s. During the odor discrimination experiment, the mice were sequentially exposed to acetaldehyde, pentanol, and heptanone. Each odor of the three odors was given three successive trials. Each trial lasted for 1 min with an inter-trial interval of 30 s. Effective sniffing was defined as the distance between the mouse's nose and Q-tip being less than 1 cm. The exploring time of the mice in each trial can be obtained via the sum of the sniffing time. 8 mice at 6-month age in each group were used in these experiments. The odor discrimination index of mice was calculated by the difference between the sniffing time of mice in the third trial of every odor and the sniffing time in the first trial of subsequent odor [42].

### Screening of potential compounds targeting SNAP25

Considering that the crystal structure of the independent SNAP25 has not been completely dissected, we used the SNAER complex containing the SNAP25 structure for the follow-up study. The PDB file containing SNARE protein information was acquired from the RCSB Protein Data Bank (PDB accession#: 3HD7) [43]. The potential active site residues of SNAP25 in the SNAER complex were predicted by the Site Finder module of the Molecular Operating Environment (MOE) software and the DoGSiteScorer module of protein.plus [44]. The structure of SNAP25 was optimized by adding hydrogen atoms and charges and prepared for subsequent virtual screening. Structure information for 1810 FDA-approved drugs was provided by the ZINC database (https://zinc20.docking.org/). Structural optimizations of small-molecule drugs, including 3D coordinates, hydrogen atoms, and Minimize Energy are done by Chemoffice calculations. First, we performed a shape-based virtual screening using Sailvina software to search for conformations in a grid space of size less than or equal to 22.5 Å, centered on *x*, *y*, *z* = 176.037, 48.139, 152.503. To eliminate false positive results, we restricted the cutoff value (total score ≤ − 6) in the docking score function. Next, we used the Induced Fit function in the DOCK module of MOE to re-optimize the virtual screening results obtained above to ensure functional binding of the drug to the target SNAP25. Functional annotation for residue information of drug-protein interactions is mainly analyzed by PLIP (Protein–Ligand Interaction Profiler, https://plip-tool.biotec.tu-dresden.de/plip-web/plip/index). Visualization and analysis of SNARE pocket prediction and ligand–protein interactions were produced using PyMOL (https://pymol.org/2/), Discovery Studio, and MOE software.

### Statistical analysis

SPSS 22.0 and OriginPro 2021b software were used for statistical data analyses. For the data of RT-qPCR, the student’s* t* test was used for comparison between the two groups. Analysis of variance (ANOVA) was used to explore the significance of the hub genes. Bonferroni corrections were applied to correct for multiple comparisons. The data of the olfactory behavior tests were analyzed using the paired samples *t* test. For correlation analysis of SNAP25 in HIP and OB/OE samples, the Pearson correlation coefficient was calculated. The statistical graph was drawn with GraphPad Prism8. Quantitative data are shown by the mean ± SEM., and *P* < 0.05 was considered statistically significant.

### Supplementary Information

**Additional file 1: Table S1.** Primer list in the RT-qPCR.

## Data Availability

The databases that support the findings of this study are publicly available in (GEO) at (https://www.ncbi.nlm.nih.gov/geo/). All data analyzed in this work are included in this article and its supplementary information files.

## Abbreviations

AD: Alzheimer’s disease
PD: Parkinson’s disease
GEO: Gene Expression Omnibus
DEGs: Differentially expressed genes
NDDs: Neurodegenerative diseases
PPI: Protein–protein interaction
HIP: Hippocampal
SN: Substantial nigra
OB: Olfactory bulb
OE: Olfactory epithelium
RT-qPCR: Real-time quantitative polymerase chain reaction
GO: Gene Ontology
KEGG: Kyoto Encyclopedia of Genes and Genomes
MOE: Molecular Operating Environment
SNARE: Soluble *N*-ethylmaleimide-sensitive fusion protein attachment protein receptor
