# Supplementary material for: SNAP25 is a potential target for early stage Alzheimer’s disease and Parkinson’s disease
Source: Eur J Med Res. 2023 Dec 6;28:570. doi: 10.1186/s40001-023-01360-8 (PMC10699008; doi:10.1186/s40001-023-01360-8)
Supplement: Supplementary file 1 — Additional file 1: Table S1. Primer list in the RT-qPCR. [file 40001_2023_1360_MOESM1_ESM.docx]

| **Gene** | **Primers** | **Sequence (5'→3')** |
| --- | --- | --- |
| SNAP25 | Forward primer | ACCAGTTGGCTGATGAGTCG |
|  | Reverse primer | CAAAGTCCTGATACCAGCATCTT |
| SYN1 | Forward primer | AGTTCTTCGGAATGGGGTGAA |
|  | Reverse primer | CAAACTGCGGTAGTCTCCGTT |
| SYT1 | Forward primer | GTGAGCGAGAGTCACCATGAG |
|  | Reverse primer | CCCACGGTGGCAATGGAAT |
| GAP43 | Forward primer | GGCCGCAACCAAAATTCAGG |
|  | Reverse primer | CGGCAGTAGTGGTGCCTTC |
| SNAP91 | Forward primer | AGCCGGTCATGTTTGCACA |
|  | Reverse primer | AGATCCGCTAATGGGTCCTTT |
| β-actin | Forward primer | ATGAACGCTACACACTGCATC |
|  | Reverse primer | CCAGTTGGTAACAATGCCATGT |

**Table S1** Primer list in the RT-qPCR
